# Convergence of Rad6/Rad18 and Fanconi Anemia Tumor Suppressor Pathways upon DNA Damage

**DOI:** 10.1371/journal.pone.0013313

**Published:** 2010-10-13

**Authors:** Hwan Ki Park, Hong Wang, Jun Zhang, Suvamoy Datta, Peiwen Fei

**Affiliations:** 1 The Hormel Institute, University of Minnesota, Austin, Minnesota, United States of America; 2 Department of Laboratory Medicine and Pathology, Mayo Clinic College of Medicine, Rochester, Minnesota, United States of America; Wayne State University, United States of America

## Abstract

Extremely high cancer incidence associated with patients with Fanconi anemia (FA) suggests the importance of the FA signaling pathway in the suppression of non-FA human tumor development. Indeed, we found that an impaired FA signaling pathway substantially contributes to the development of non-FA human tumors. However, the mechanisms underlying the function of the FA pathway remain less understood. Using RNA interfering approach in combining with cell proliferation and reporter assays, we showed that the function of FA signaling pathway is at least partly mediated through coupling with hRad6/hRad18 signaling (HHR6 pathway). We previously reported that FANCD2 monoubiquitination, a hallmark of the FA pathway activation, can be regulated by HHR6. Here we found that hRad18 can also regulate activation of the FA pathway. More importantly, we found that FANCD2 is capable of modulating activity of DNA translesion synthesis polymerase eta, an effector of HHR6 pathway. These results provide novel insights into how the FA pathway is intertwined with HHR6 pathway to maintain chromosomal stability and suppress the development of human cancer, representing an important conceptual advance in the field of FA cancer research.

## Introduction

Our genome is constantly bombarded by both exogenous and endogenous genotoxic stresses, eventually leading to DNA damage. Well-orchestrated cellular responses to DNA damage are absolutely required to maintain genome stability and thus prevent diseases [1]. The coordinated responses will either eliminate damaged cells or repair the damage to ensure a normal cell growth [2]. To date, homologous recombination, non-homologous end joining, nucleotide excision repair, base excision repair, translesion synthesis, and DNA-crosslink repair are known repair responses to DNA damage, among which DNA-crosslink repair is attributed to all other damage repair processes described [3]. In response to DNA crosslinks, a mammalian DNA damage signaling pathway, called Fanconi Anemia (FA) pathway, is activated [3], [4]. This signaling pathway is determined by similar symptoms displayed from at least 13 or 14 complementation groups of FA [5]–[7], which is a rare human genetic disease featured with severe bone marrow failure, many congenital defects, and an extremely high cancer incidence [3], [4]. Within the FA pathway, the multi-FA protein complex can act as an E3 ubiquitin ligase to monoubiquitinate FANCD2 and its paralog FANCI, and the monoubiquitinated FA proteins then function in concert with other known or unknown proteins to repair DNA damage and maintain chromosomal stability [3], [8]. FANCD2 monoubiquitination thus appears to be a measure of the activation of this DNA-crosslink damage response pathway. *Yeast* or *Bacteria* can not survive through one single DNA-crosslink if not repaired [4]. In humans, impaired FA signaling was recently identified to be an important factor in promoting the development of non-FA human cancer [9]. However, it still remains unknown as to how the FA signaling pathway or FANCD2 protein functions.

We previously found that HHR6 (human homolog of yeast rad6), a major player in HHR6 pathway signaling upon DNA damage, can regulate FANCD2 monoubiquitination [10]. In HHR6 pathway, also called postreplication repair (PRR), a set of complex DNA replication recovery/damage tolerance processes permit DNA synthesis over a damaged template. This damage response pathway is composed of two subpathways: error-prone translesion synthesis (TLS) [11] or an error-free system [12], involving downstream reinitiation followed by gap-filling through recombinational events. The key components in TLS are low fidelity DNA polymerases specialized in lesion bypass, which are evolutionally conserved. In human, there are at least four DNA polymerases belonging to Y superfamily (pol η, pol κ, pol ι and REV1)[12] as well as a few from other polymerase families. The existence of these DNA polymerases with very low fidelity suggests that their participation in genome replication needs to be carefully regulated. But molecular events that regulate these TLS DNA polymerases in humans remain unclear. Studies in *S. cerevisiae* suggest that post-translational modification of proliferating cell nuclear antigen (PCNA), an accessory factor of replicative DNA polymerases, is important for the switch from the replicative polymerase to a TLS polymerase [11], [13]–[15]. PCNA monoubiquitination under the control of Rad6/Rad18 is believed to provide a platform for both error-prone and error-free TLS events possibly to occur. Here we found that hRad18 can also regulate FANCD2 activation, and that human TLS DNA polymerase eta (pol η) can be regulated by FANCD2, indicating the function of the FA pathway is at least partly mediated through coupling with the HHR6 pathway.

## Results

### Deficient hRad18 Impairs FANCD2 Monoubiquitination/Activation

**Figure 1:**
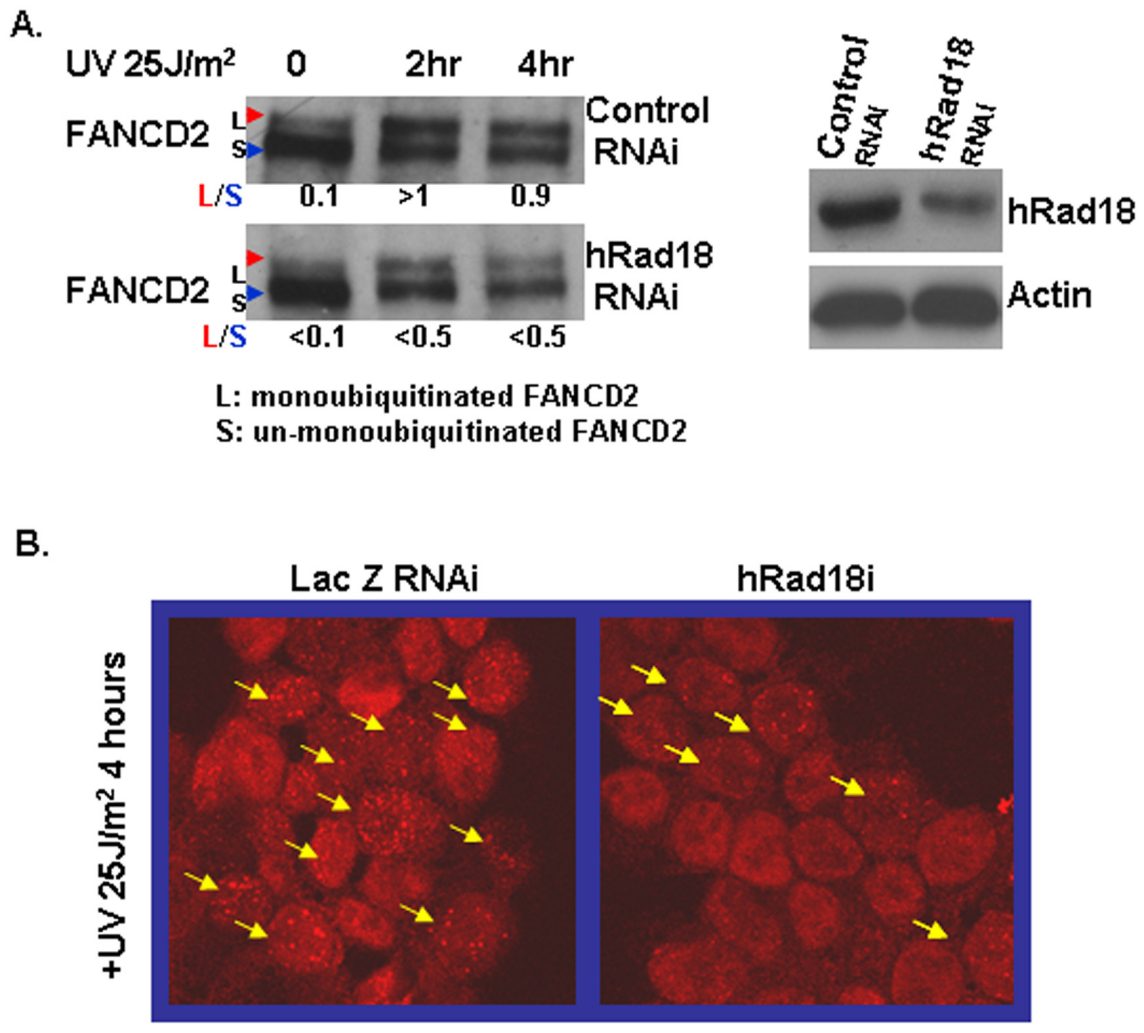
Down-regulation of hRad18 leads to compromised FANCD2 monoubiquitionation and focus formation in response to DNA damage. Populations of U2OS cells either transfected with hRad18 RNAi oligos or non-specific control oligos. 18 hours posttransfection, cells were split, and followed by 25J/m^2^ UV treatment. In a population of hRad18 downregulated cells, the ratio of monoubiquitinated FANCD2 over unmonoubiquitinated FANCD2 was decreased upon UV treatment at both time points examined (A). FANCD2 focus formation was also decreased in hRad18-silenced cells, and images shown in (B) were taken from these cells at time point of 4 hours [FANCD2 is a nuclear protein and forms foci (marked with yellow arrows) when monoubiquitinated] [(similar results were found in Hela cells (not shown)].

The fact that FANCD2 is activated during DNA synthesis or upon DNA damage suggests the existence of close interplays between FANCD2 and players in the processes of DNA synthesis and DNA damage repair. The hypersensitivity to DNA-crosslinking agents, such as UV-irradiation, inherent to cells derived from patients with FA or XPV (Xeroderma Pigmentosum Variant) [16], or to yeast deficient in Rad6 [17], [18], further indicates specific links among FANCD2, pol η, and human homologs of yeast Rad6 (HHR6). To uncover the mystery of FANCD2 function, we decided to reveal these potential signaling links. Indeed, we found that HHR6 can regulate FANCD2 activation/monoubiquitination [10]. Here we questioned whether hRad18 can also regulate FANCD2 monoubiquitination upon DNA damage, becasue HHR6 cooperates with hRAD18 to modulate the function of proteins involved in DNA damage responses [19]–[21]. Using RNA interfering (RNAi) approach, we found cells carrying different levels of hRad18 protein displayed altered levels of monoubiquitinated FANCD2 following exposure to UV (Fig. 1A) or mitomycin C (MMC) (not shown), suggesting the involvement of hRad18 in the regulation of FANCD2 monoubiquitination. Moreover, FANCD2 focus formation, an additional measurement for FANCD2 activation/monoubiquitination was also found to be compromised in corresponding UV-treated cells carrying a low level of hRad18 expression (Fig. 1B). Taken together, upon DNA damage, FANCD2 activation is at least partly regulated by hRad18.

### Downregulation of hRad18 leads to a similar cell sensitivity to that of FANCL

**Figure 2:**
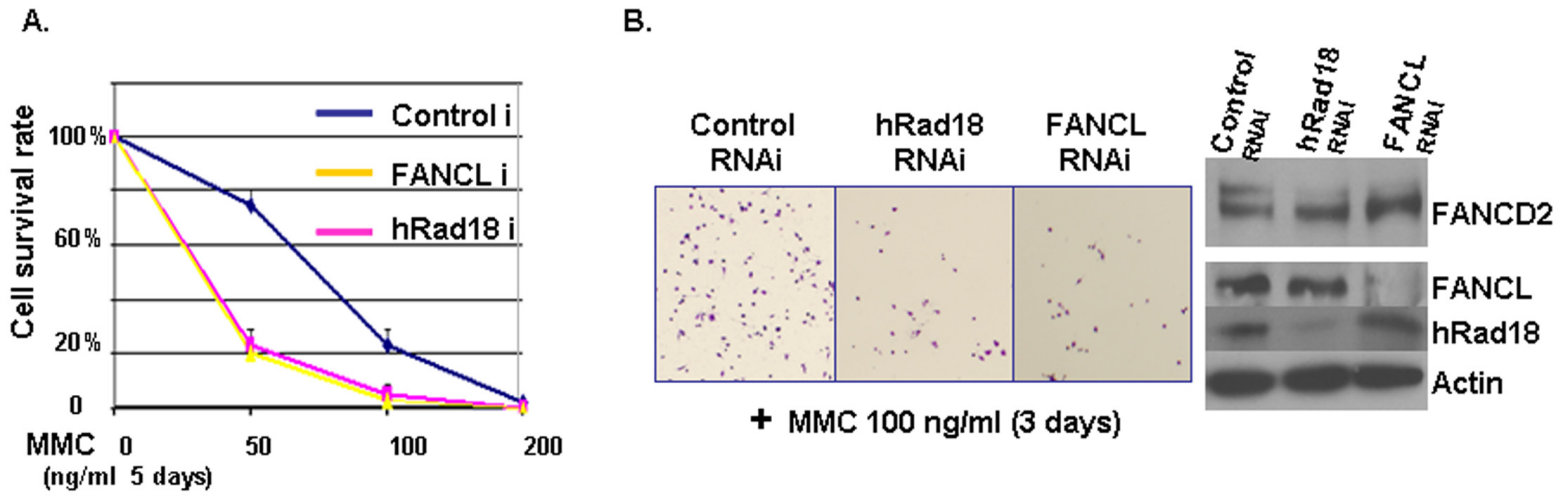
Silencing hRad18 and FANCL Lead to a Similar Mitomycin C Sensitivity. Equal populations of U2OS or Hela cells, transfected with RNAi oligos for non-specific control (Control RNAi), targeting hRad18 (hRad18 RNAi), or targeting FANCL (FANCL RNAi), were treated with MMC at 50, 100, and 200 ng/ml for 5 days. Surviving cells were counted, and plotted for the survival rate curve. Cells, either carrying down-regulated hRad18 or FANCL, showed a similar survival rate (pink and yellow lines) (A). The images of U2OS cells treated with 100 ng/ml MMC are shown in the right along with RNAi targeting efficiency (Western blotting). Down-regulated hRad18 or FANCL by corresponding RNAi oligos interfered with FANCD2 monoubiquitination and affected cell survival (Results generated from Hela cells are all similar to ones shown here).

FANCD2 monoubiquitination is an essential event in the FA signaling transduction. Cells deficient in FANCD2 monoubiquitination, such as FA cells, are hypersensitive to interstrand DNA cross-linking (ICL) agents [22]. To further verify hRad18 regulation of FANCD2, we tested whether the deficient monoubiquitination of FANCD2 triggered by a lower level of hRad18 expression also confers similar cell sensitivity to DNA crosslinking agents as the one triggered by deficient FA genes. Populations of U2OS or Hela cells, transfected with either non-specific RNAi oligos, specific RNAi oligos targeting hRad18 or FANCL, were treated with a series of different mitomycin C (MMC) concentrations (50 ng/ml–200 ng/ml) for 5 days to examine cell survival ability. Both cell lines showed a similar decline in survival rate with an exception about 5% more Hela cells survived at each drug dose tested respectively (Fig. 2A, and data not shown). Images of cell density for U2OS (Fig. 2B) or Hela cells (not shown) support their declined cell growth rates. Moreover, cell populations treated with RNAi oligos against either FANCL or hRad18 exhibited a similar reduction in cell growth following MMC treatment as compared to cells treated with control non-specific RNAi oligos. These results indicate that, although it remains unknown the manner, by which hRad18 regulates FANCD2 monoubiquitination, hRad18 and FA complex appear to be capable of sharing a common downstream target, FANCD2, in the responses of cells exposed to MMC tested here.

### FANCD2 is a Regulator of DNA Translesion Synthesis Polymerase Eta

**Figure 3:**
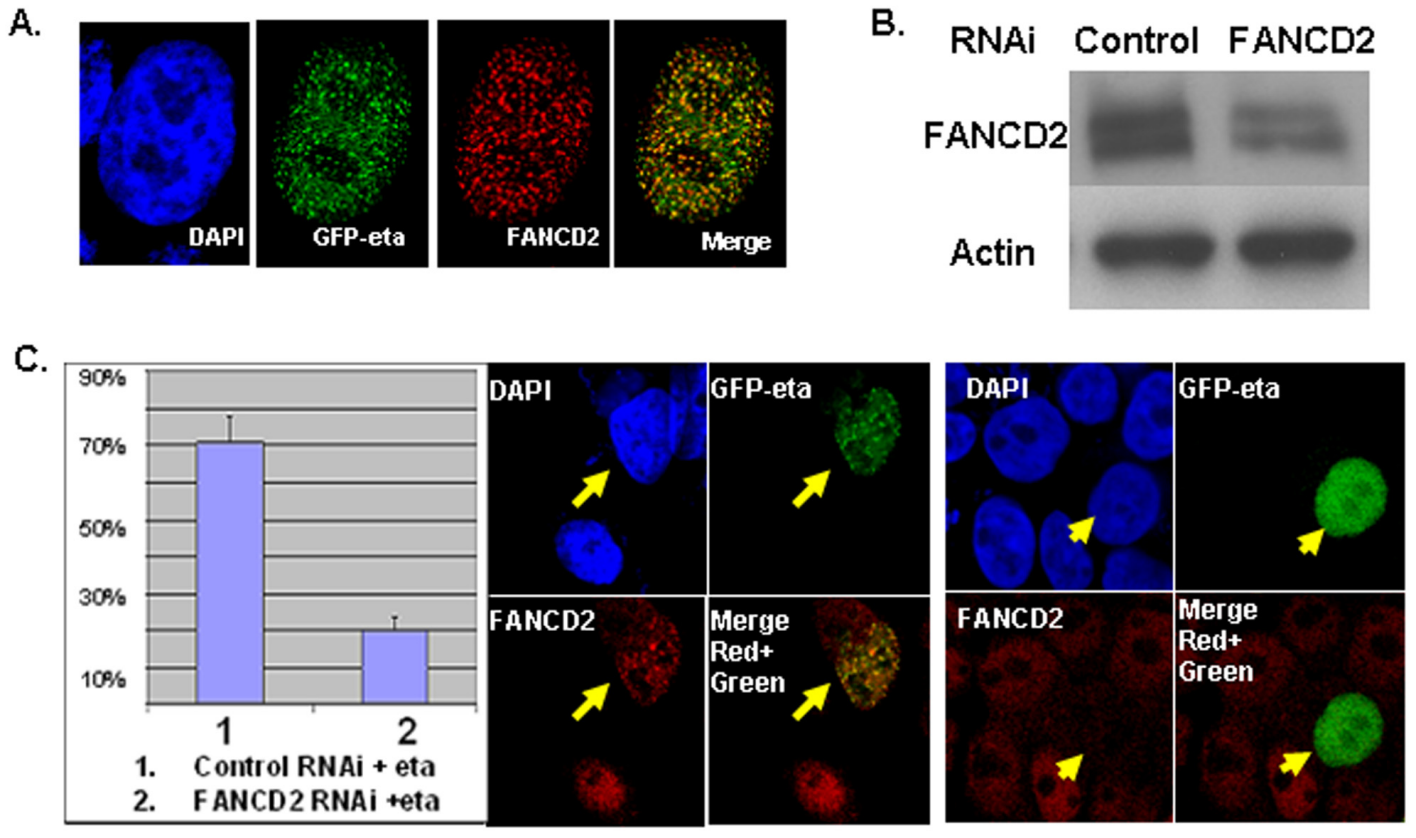
Polymerase Eta Focus Formation Depends on FANCD2. **A. FANCD2 foci colocalize with the foci of polymerase eta.** U2OS cells were split at 30% confluence the day before transfection. At 24 hours after transfection, cells transfected with GFP-eta plasmid or empty vector control were treated with UV (25 J/m^2^), and were collected 8 hours after UV exposure. Subsequently, these cells were fixed for a standard immunofluorescent study by using antibodies against human FANCD2. FANCD2 foci can colocalize with the green foci, which result from the exogenous GFP-fused polymerase eta. (We did not observe any green foci or a clear green signal in the nuclei of the cells transfected with empty vector control - data not shown.) (The yellow dots show the colocalization.) (**B, C**) **Knocking Down FANCD2 Proteins leads to a Decreased Percentage of Polymerase Eta Focus Formation,** Cells were first transfected with control RNAi oligos or oligos specifically targeting FANCD2. These cells were then transfected with GFP-pol η at 24 hour point after RNAi oligo transfection. These doubly transfected cells were next treated with UV (20 J/m^2^) 24 hours after transfection and were collected 8 hours after UV treatment. Subsequently, a standard immunofluorescence assay was performed. Total levels of FANCD2 protein were detected in cells with control RNAi oligos and FANCD2 RNAi oligos (B). The ratio of green focus formation over total green cells was decreased in cells expressing FANCD2 at a lower level (C left). The typical image of two given cells expressing FANCD2 protein at a high or low level, showed focus formation or non-focus formation of pol η, respectively. (Standard deviation was generated from three separate experiments.) (The arrows mark the same individual cell for each panel, and the red stain represents FANCD2 protein; green for pol η; blue for nuclear staining; and yellow for the merge of red and green.).

In response to DNA damage, the signaling cascade initiated by HHR6-hRAD18 is known as HHR6 pathway/PRR [23], within which PCNA is a mediator of HHR6/hRad18 to regulate the function of DNA-translesion synthesis polymerases including pol η. We have found that FANCD2 can be regulated by HHR6 [10] as well as by hRad18 (Fig. 1), suggesting FANCD2 may act as an additional mediator of HHR6/hRad18 to regulate downstream events of HHR6 pathway/PRR. Furthermore, the similar DNA damage sensitivity displayed by cells carrying deficient FANCD2 or pol η indicate a functional link between FANCD2 and pol η. We thereby anticipated a potential functional relation between FANCD2 and pol η, specifically between two protein focus formations given that both proteins are known to form foci in response to DNA damage [24], [25]. Indeed, we found focus colocalization between FANCD2 and pol η in cells following UV exposure (Fig. 3A). To this point, we further asked whether FANCD2 plays regulatory roles for the formation of pol η foci. We created a cellular system expressing FANCD2 protein at different levels by transfecting U2OS cells with RNAi oligos targeting FANCD2 or non-specific for controls (Fig. 3B). We then re-transfected these cells with plasmid containing GFP-pol η, followed by analyzing the effects of FANCD2 protein expression levels on focus formation of pol η. We found that the percentage of green focus formation over total green cells is higher in cells transfected with control RNAi oligos, in which the levels of FANCD2 are normal, compared to cells with FANCD2 RNAi oligos leading to a lower level of FANCD2 protein (Fig. 3B and 3C). Therefore, FANCD2 is able to regulate the focus formation of pol η. Similar results were obtained in FA patient cells PD20 (FANCD2-/-), compared to relevant control cells carrying functional FANCD2 (data not shown). These results indicate that, in response to DNA damage, pol η may act as a functional downstream target of FANCD2 and thus, at least partly, mediate FANCD2 function in maintaining chromosomal stability.

### FANCD2 Can Modulate the Activity of DNA Translesion Synthesis

**Figure 4:**
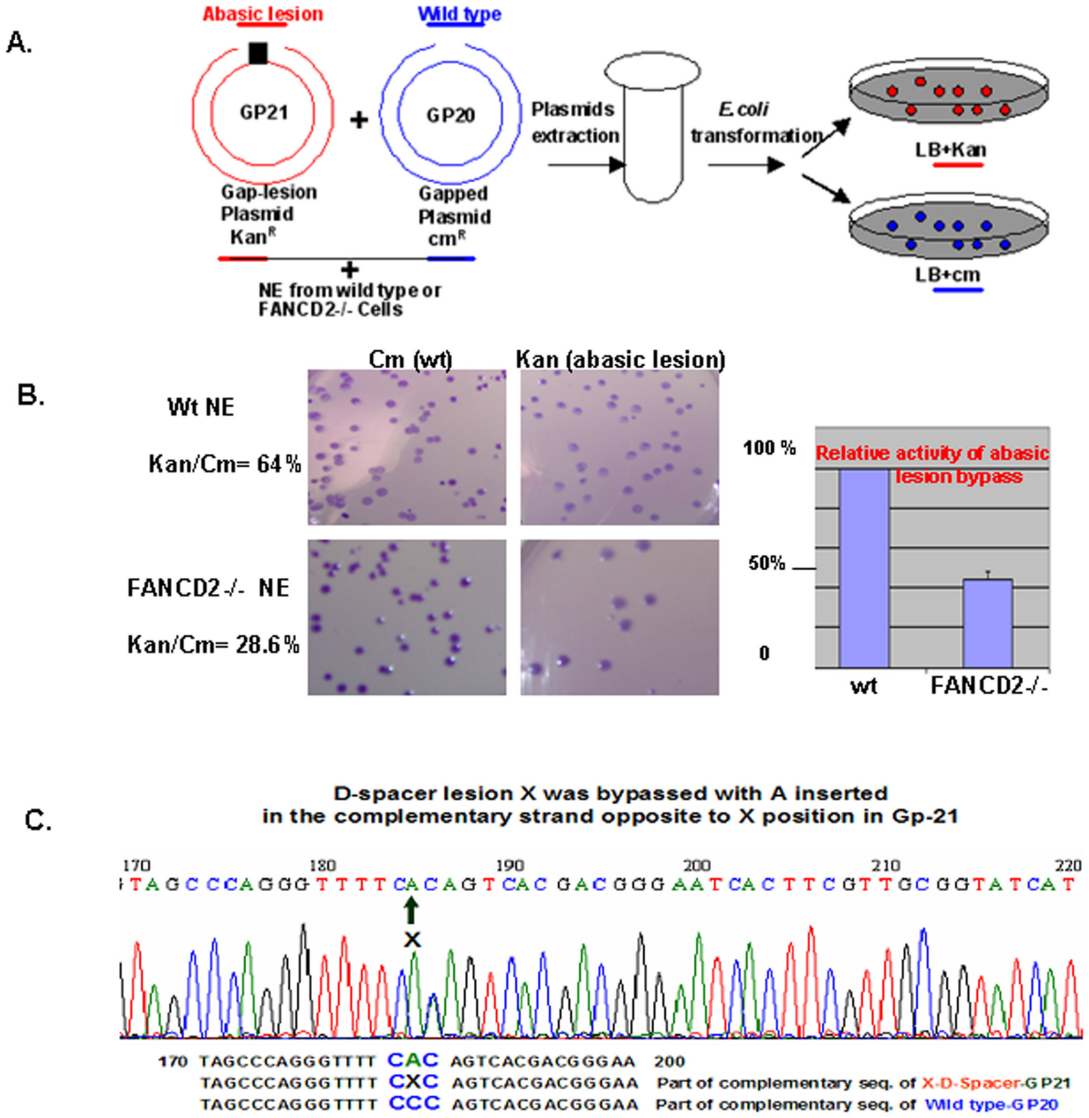
FANCD2 Regulates DNA Translesion Synthesis Activity. (**A**) **Schematic representation of in vitro abasic lesion bypass reporter assay.** The number ratio of colonies grown in a *Kan-containing LB* plate over those grown in a correspondent *Cm* plate can be influenced by the enzymatic activity of pol η. An increased activity of polymerase eta will promote lesion-gap filling; a decreased activity of polymerase eta will inhibit lesion-gap filling. Therefore, this ratio change will be the readout for the regulation on enzymatic activity of pol η. (**B**) **The bypass activity of abasic lesion is decreased in FANCD2-/- cells (PD20, derived from FA patient).** 50 ng of each gap plasmid (wt or abasic lesion) and 50 µg of NE with or without FANCD2, prepared from PD20+D2 or PD20 cells 6 hr following 35 J/m^2^ UV treatment, were used for the assay. Compared to FANCD2 proficient NE, the lesion gap filling capacity was decreased in FANCD2 deficient NE, from which the extracted plasmids lead to a low ratio of bacterial colonies containing lesion plasmid over the ones carrying wild type plasmid (28.6%). The relative bypassing activity is downregulated more than 50% in FANCD2 deficient cells compared to FANCD2 proficient cells, which was plotted with three separated experiments. Images of bacterial colonies shown were from one of three studies. **C. Sequence verification of GP-21 plasmid isolated from bacterial colonies grown on the LB Kan plate.** About 10 colonies were randomly picked up from the *Kan* plate shown, and the isolated plasmids were checked with a correct size. Two of them again were randomly picked up and sent for sequencing. The sequence results were the same. ***D*** (abasic)-spacer lesion was bypassed with ***A*** inserted in the complementary DNA strand opposite to X lesion position in GP-21 plasmid.

HHR6 pathway/PPR is initiated upon the stalled replication forks resulting from many types of DNA lesions including the abasic one. Translesion synthesis DNA polymerases can synthesize DNA through lesion templates to prevent the collapse of the stalled replication-forks [11], [26]. Among these lesion bypassing DNA polymerases, pol η can synthesize DNA along templates carrying damages including the abasic lesion by inserting A or G into the newly synthesized complementary strand [23], [27], [28]. A well-defined abasic bypass reporter system [23] thus can provide a functional readout for FANCD2 regulation of pol η. We expected that the abasic-bypass reporter activity should be different in cells carrying different levels of FANCD2 protein expression, as suggested by data shown in Figure 3. To reveal this potential effect of FANCD2, we performed reporter assay of abasic translesion synthesis in vitro by using nuclear extracts (NEs) with deficient or proficient FANCD2. As illustrated in Figure 4A, the reporter mixture was mixed with NEs of FA patient cells carrying FANCD2-/- (PD20 cells) or FANCD2+/+ (complemented PD20 cells), respectively in the DNA synthesis buffer containing dNTPs, and incubated for 3 hours at room temperature. Subsequently we extracted plasmids and followed procedures described previously [23]. We found that abasic bypass activity was dramatically decreased in NEs without FANCD2, compared to the NE carrying FANCD2 (Fig 4B). We also performed in vivo reporter assay by transfecting plasmids into U2OS cells carrying different levels of FANCD2 expression through RNAi approach, followed by similar procedures used for in vitro assay with an exception of extracting plasmids out of cells rather than from the reaction mixtures. The results were consistent with ones derived from the in vitro assay (data not shown). Clearly, FANCD2 plays an essential role in the regulation of, at least, bypassing abasic DNA lesion and appears to be a novel regulator of DNA translesion synthesis polymerase eta.

## Discussion

**Figure 5:**
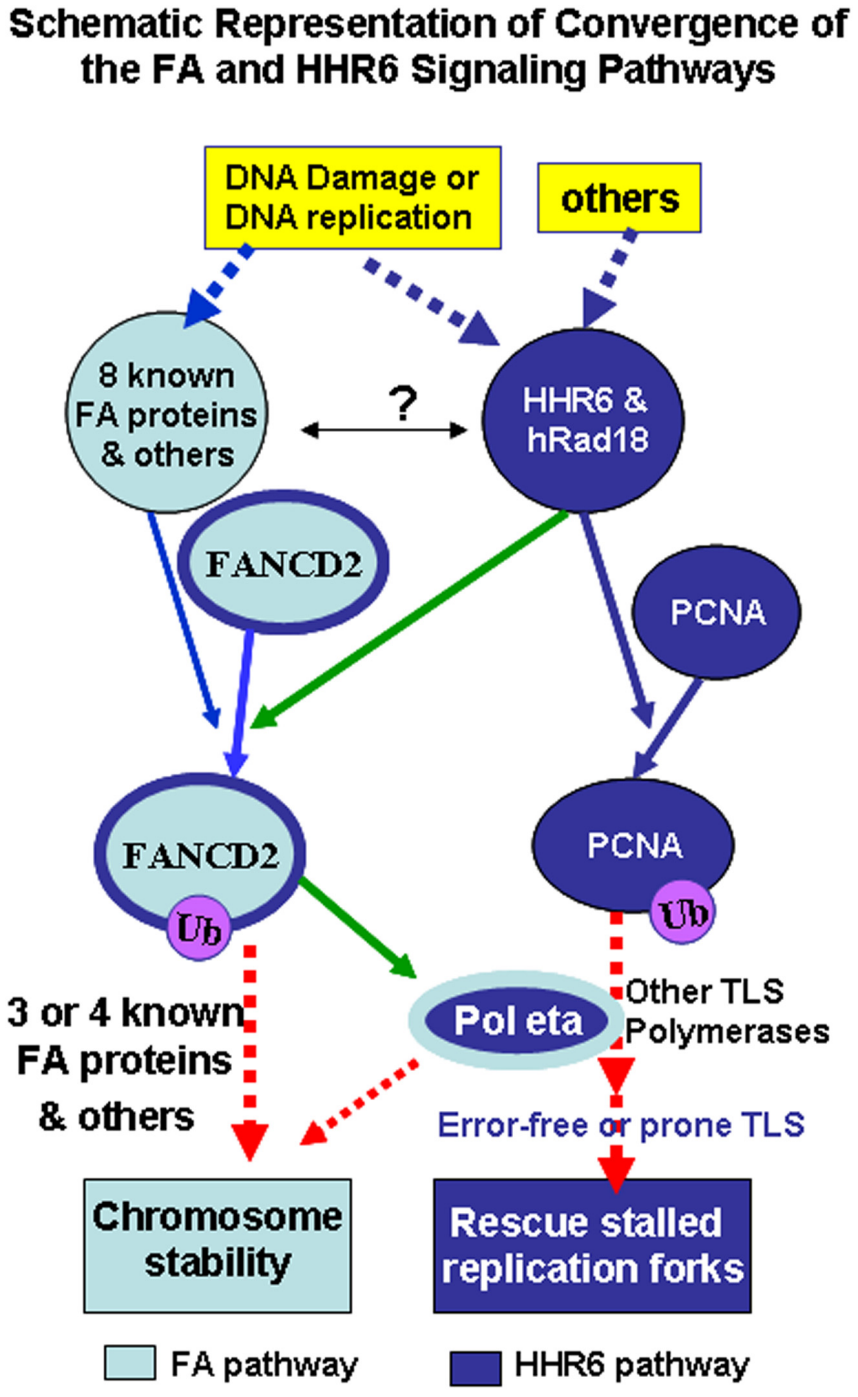
Schematic Representation of Convergence of the FA and HHR6 Pathways. Our work (green lines) indicates that HHR6/HRad18 can regulate FANCD2 monoubiquitination, a hallmark of the activation of the FA pathway (marked with light blue color); and FANCD2 can modulate the activity of polymerase eta, an effector of HHR6 pathway (highlighted with dark blue color). Upon DNA damage, FANCD2 may partly mediate the function of HHR6/hRad18 in HHR6 pathway/PRR; pol eta can as well mediate a part of FANCD2 function in maintaining chromosomal stability in the FA signaling. It waits for future studies to further define the integration of the FA and HHR6 pathways upon genotoxic stresses, such as whether and how the FA complex and hRad18 depend on each other in terms of regulation of FANCD2 monoubiquitination?

The similar DNA damage sensitivity between systems carrying either deficient HHR6 or improper FA signaling prompted us to explore a possible signaling link between HHR6 and FA pathways. Indeed, we found that not only HHR6 [10] but also hRad18 are capable of regulating the activation of FANCD2 (Fig 1). More importantly, we found FANCD2 can regulate translesion synthesis (TLS) DNA polymerase eta (Fig. 3, 4), which appears to be a first protein to be indentified acting as a mediator of FANCD2 function. This finding is consistent with the concept long-proposed in the field of FA study that FA proteins may play roles in TLS [3], [4], [29]. Unlike many known TLS enzymes, pol η mostly synthesizes DNA accurately upon the lesion template [12], [30], as demonstrated by XPV resulting from a mutated pol η [16], [31]. We believe chromosomal stability maintained by FANCD2/FA pathways is at least partly attributed to the function of pol η; pol η may as well be a potential candidate FA-like or FA-related gene for those unclassified FA patients. Furthermore, the regulation of pol η by FANCD2 may indicate a part of FANCD2 broader effects, rather than strictly functioning in the FA signaling. The majority of FA core complex genes are completely absent in many eukaryotic species that do contain orthologues of FANCD2 [3], [4]. This difference also suggests the additional function that FANCD2 may have. In fact, FANCD2 has been suggested to participate in a separate signaling pathway that is activated by ATM in response to ionizing radiation [32], and it was recently also found to induce apoptosis [33]. Collectively, our study provides a framework for our understanding of maintaining chromosomal stability through converging two DNA damage response pathways (Fig. 5), in which many questions await answers, such as how HHR6/hRad18 regulates the activation of FANCD2/the FA pathway, directly or indirectly? Or simply, Pol η is regulated by monoubiquitinated FANCD2, un-monoubiquitinated FANCD2, or both? We believe our ongoing and future studies, and those of others will provide refined mechanisms underlying the integration of HHR6 and FA pathways in maintaining genomic stability (Fig. 5).

## Materials and Methods

### Cell lines and Chemicals

All cell lines were obtained from the American Type Culture Collection (ATCC), with an exception of specially engineered ones. Mitomycin C was purchased from Sigma.

### Cell survival assay

Equal numbers of U2OS or Hela cells were seeded in 60 mm dishes one day prior to transfection with control non-specific RNAi oligos or RNAi oligos against hRad18, or FANCL. Thirty-six hours posttransfection, cells were treated with, 50 ng/ml, 100 ng/ml, or 200 ng/ml MMC for 5 days. The number of surviving cells was scored, and cell numbers were plotted as cell survival curves (cell numbers of the samples treated with the drug were normalized to the cell numbers of the untreated control sample. Each drug dose was tested in triplicate). The shown images were RNAi oligo-transfected cells, which were treated with 50 ng/ml MMC for 3 days.

### Reporter plasmids, Nuclear Extract Preparation, and in vitro DNA Synthesis

GP21 abasic plasmid (*kan*-resistance) and GP20-gap plasmid (*cm*-resistance) were constructed as described [23]. The GP21 abasic primer: 5′-ACCGCAACGAAGTGATTC CCGTCGTGACTG**X**GAAAACCCTGGGCTACTTGAACCAGACCG -3′; GP-20 gap primer: 5′- ACCGCAACGAAGTGATTCCCGTCGTGACTG**G** GAAAACCCTGGG CTACTTGAACCAGACCG -3′; TLS (XmnI) primer: 5′- GGA ATC ACT TCG TTG -3′; and TLS (BstXI) primer: 5′- CTG GTT CAA GTA GCC -3′ (**X** is an abasic site). Nuclear Extracts (NE) were prepared from PD20 and PD20 + DANCD2 cells by using Kit#78833 (Thermo Scientific). The in vitro assay was set up by mixing GP20 (50 ng), GP21(50 ng), and NE (50 µg) in Tris-HCl buffer (40 mM, pH 7.5) including MgCl_2_ (5 mM), dithiothreitol (1 mM), bovine serum abumin (100 µg/ml), 10% glycerol, and dNTP (100 µM). The mixture was incubated at room temperature for 3 hours, followed by phenol chloroform extraction and precipitation of plasmid DNA. Subsequently, the extracted plasmid mixture was transformed into DH5α competent bacteria and equally spread on *cm (chloramphenical)* and *kan (kanamycin)* containing LB agar plates.

### Immunostaining, Western blotting, and siRNA oligonucleotide transfection

These techniques were performed essentially as described [9], [10], [34]. HRad18 antibody was purchased from Santa Cruz and used at a dilution rate of 1∶500 for Western blotting. All RNAi ologos were purchased from Dhmarcom, targeting hRad18 (cttgctgtg tgactgtcac), FANCL (gacaagagctgtatgcact) [35], or FANCD2 (ccaggaagcaaccactttc).
